# Optomechanical terahertz detection with single meta-atom resonator

**DOI:** 10.1038/s41467-017-01840-6

**Published:** 2017-11-17

**Authors:** Cherif Belacel, Yanko Todorov, Stefano Barbieri, Djamal Gacemi, Ivan Favero, Carlo Sirtori

**Affiliations:** 1 Laboratoire Matériaux et Phénomènes Quantiques, Université Paris Diderot, Sorbonne Paris Cité, CNRS-UMR 7162, 10 rue Alice Domont et Léonie Duquet, 75013 Paris, France; 2 Present Address: IEMN (Institute of Electronics, Microelectronics and Nanotechnology), University of Lille and CNRS, UMR 8520, 59652 Villeneuve d’Ascq, France

## Abstract

Most of the common technologies for detecting terahertz photons (>1 THz) at room temperature rely on slow thermal devices. The realization of fast and sensitive detectors in this frequency range is indeed a notoriously difficult task. Here we propose a novel device consisting of a subwavelength terahertz meta-atom resonator, which integrates a nanomechanical element and allows energy exchange between the mechanical motion and the electromagnetic degrees of freedom. An incident terahertz wave thus produces a nanomechanical signal that can be read out optically with high precision. We exploit this concept to demonstrate a terahertz detector that operates at room temperature with high sensitivity and a much higher frequency response compared to standard detectors. Beyond the technological issue of terahertz detection, our architecture opens up new perspectives for fundamental science of light–matter interaction at terahertz frequencies, combining optomechanical approaches with semiconductor quantum heterostructures.

## Abstract (web summary)

Achieving fast, sensitive and room temperature detection of terahertz waves remains a formidable scientific and technological challenge. Here, the authors propose a compact terahertz device combining concepts from metamaterial resonators, optomechanics and semiconductor nanotechnology.

## Introduction

The terahertz (THz) spectral domain offers myriads of applications spanning chemical spectroscopy, medicine, security and imaging^1^. Terahertz waves are fascinating owing to their peculiar position in the electromagnetic spectrum, bridging microwaves and infrared light. Since metals do not suffer prohibitive optical losses in this spectral range, one can rely on metallic structures such as metamaterial resonators^2–6^, metallic waveguides^7, 8^ and antennas^9, 10^ that are able to concentrate electromagnetic energy into very small volumes. The combination of such electromagnetic architectures with low-dimensional semiconductor hetero-structures or graphene has recently opened new venues for fundamental studies of light–matter interactions^3, 4, 7, 8^, as well as novel detector concepts^5, 6, 10, 11^. The detection problem is particularly severe in the terahertz range, where widespread technology relies on slow thermal detectors, and room temperature operation is never compatible with high speed and high sensitivity^12, 13^.

Terahertz science and technology could benefit from optomechanical approaches, which harness the interaction of light with miniature mechanical resonators^14, 15^. So far, optomechanics has mostly focused on the optical and microwave domains, leading to new types of quantum experiments^16–18^ and to the development of optical-microwave converters^19–21^.

Here we demonstrate a THz detector based on the combination of concepts derived from metamaterial resonators, optomechanics and semiconductor nanotechnology. Our device consists of an integrated meta-atom terahertz resonator with a flexible part acting as a mechanical oscillator. Free-space terahertz photons are collected by the resonator and induce high-frequency currents and charges that, in turn, couple to the mechanical degrees of freedom. The resulting mechanical motion is read out optically, allowing our device to function as a compact and efficient terahertz detector at room temperature. Furthermore the device responds at high frequencies (>10 MHz), well beyond the cut-off frequencies of Golay cells, pyroelectric detectors and cryogenic semiconductor bolometers^12, 13^. Our experiments unambiguously reveal an instantaneous detection mechanism arising from a nano-scale Coulomb interaction, with a noise equivalent power that is potentially frequency independent. Alongside this effect, our compact geometry allows for an uncooled bolometric detection^22^ with extremely short heat diffusion times, on the order of few microseconds. Our device concept is thus qualitatively different from metamaterial bolometers demonstrated previously^6, 7^, where the metamaterial resonators act as passive absorbers on top of bulk bilayers, with typical response times on the order of milliseconds.

## Results

### Device and characterizations

**Fig. 1:**
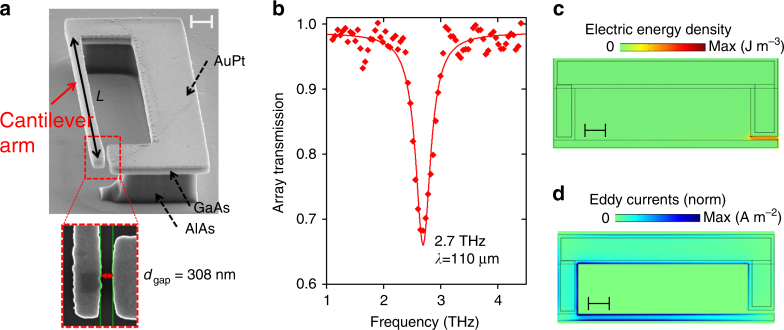
Optomechanical terahertz resonator and optical characterizations. **a** Side view of the split-ring resonator (SRR) obtained with a scanning electron microscope. The blown-up image shows the capacitive gap between the cantilever arm and the rest of the SRR. The scale bar indicated in the upper right corner of the image is 1 µm wide. **b** Far-infrared transmission spectrum of an array of identical SRRs. The dots are experimental measurements and the line is a Lorentzian fit. **c** Distribution of the electric energy density in the SRR, obtained with a finite difference domain simulator. **d** Illustration of the normalized THz eddy currents induced in the metal layers at resonance. The scale bar indicated in **c**, **d** is 2 µm wide

Our device is shown in Fig. 1a and consists of an asymmetric terahertz split-ring resonator (SRR), obtained by depositing a metal pattern on a GaAs (320 nm)/Al_0.8_Ga_0.2_As layered semiconductor structure (see Methods). By using standard semiconductor etching technology, the narrow arm of the resonator has been processed into a cantilever with a high aspect ratio (length *L* = 15.7 µm, width *w* = 444 nm and thickness *t* = 470 nm, including 320 nm of GaAs and 150 nm of gold layers). As shown in the blown-up part of Fig. 1a, the free end of the cantilever forms an arm of a 308 nm wide capacitive gap (*d*_gap_) of the SRR.

Figure 1b displays the SRR THz response measured in transmission spectroscopy with a Fourier transform interferometer and a cooled germanium bolometer (Methods). In order to increase the amplitude of the transmission feature, we used a dense array of nominally identical SRRs^9, 23^. The SRR resonance appears in the spectrum of Fig. 1b as a Lorentzian dip with a central frequency *ω*_THz_/2*π* = 2.7 THz and a quality factor *Q*_THz_ = 8.4. In Fig. 1c, we plot the electric energy density provided by finite element method simulations. The electric energy is strongly localized in the SRR gap, allowing the excitation of Coulomb forces acting on the cantilever. In Fig. 1d, we also plot the eddy currents induced in the SRR, which turn out to play an important role for the photothermal effects in the structure, as explained further.

**Fig. 2:**
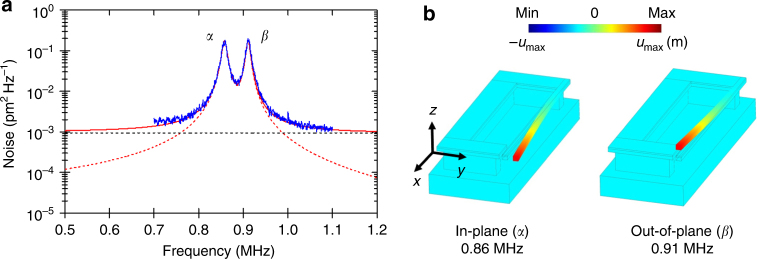
Characterization of the nanomechanical modes. **a** Blue curve: radio frequency spectra of the room temperature cantilever Brownian motion, displaying two fundamental mechanical resonances, labelled *α* and *β*. The red curve is a fit resulting from the sum of the spectral noise for two harmonic oscillators (red dashed curve) added to the noise floor of the balanced photo-diode unit (black dashed curve). **b** Numerical modelling of the amplitude of deformation *u*_max_ for each mechanical mode. The *α* resonance corresponds to the cantilever oscillations in the *Oxy* plane of the SRR and the *β* resonance corresponds to oscillations in the *Oxz* plane

To characterize the mechanical modes of the cantilever, we focus a near-infrared (NIR, *λ* = 940 nm) laser beam at its end through a microscope objective (MO). The Brownian motion of the cantilever produces intensity fluctuations in the reflected beam that are recorded as the signal difference between two balanced silicon photo-diodes (PhDs) connected to a spectrum analyser (SA). In Fig. 2a, we show the resulting radio frequency (RF) spectra of the fundamental in-plane (*α*) and out-of-plane (*β*) flexural modes, visualized in Fig. 2b. The direction of oscillation is the *y*-axis for the *α* mode and the *z*-axis for the *β* mode, using the coordinate system defined in Fig. 2b. The corresponding mechanical frequencies and quality factors are, respectively, *f*_*α*_ = 0.86 MHz, *Q*_*α*_ = 70 and *f*_*β*_ = 0.91 MHz, *Q*_*β*_ = 94. The Brownian motion is well fitted by the analytical expression of the noise spectral power density *S*_*yy*_(*f*) + *S*_*zz*_(*f*) from a dumped mechanical oscillator model^15^, taking into account the detection noise floor. The knowledge of the cantilever dimensions and composition allows determining its effective mass *m*_eff_ = 8.5 pg, and hence a known value for the room temperature peak noise spectral density *S*_*yy*,*zz*_(*f*_*α*,*β*_) = 2*k*_B_ *TQ*_*α*,*β*_/*m*_eff_(2*πf*_*α*,*β*_)^3^ for each resonance (*k*_B_ is the Boltzmann constant and *T* is the temperature). This permits a precise calibration of the cantilever displacements measured with the present technique.

### Coulomb driving force

When the structure is resonantly excited by an incident terahertz radiation, a dynamic distribution of charges with opposite signs appears on both sides of the gap, yielding strong localization of electric energy, as shown in Fig. 1c. This results into a quasi-static Coulomb force that attracts the cantilever towards the opposite end of the gap, setting it in motion. The Coulomb attractive force will have a stronger effect on the in-plane (*α*) mechanical mode, oscillating along the *y*-axis, and is described by the following equation of motion, derived from an effective capacitor on spring model (Supplementary Note 1):

1 $$\frac{{d^2y}}{{dt^2}} + \frac{{\omega _\alpha }}{{Q_\alpha }}\frac{{dy}}{{dt}} + \omega _\alpha ^2y = - \frac{{W_{{\mathrm{eTHz}}}(t)}}{{m_{{\mathrm{eff}}}}}\left. {\frac{{d\,{\mathrm{ln}}\,C_{{\mathrm{eff}}}}}{{dy}}} \right|_{y = d_{{\mathrm{gap}}}}$$

Here *C*_eff_(*y*) is the effective capacitance of the charge distribution of the terahertz mode, and *W*_eTHz_(*t*) is the total time dependent electric energy stored in the SRR. Note that the electric energy oscillates in the THz range, *W*_eTHz_(*t*) ~ cos^2^(*ω*_THz_ *t*), i.e. six orders of magnitude higher than the mechanical frequency *ω*_*α*_, a situation reminiscent of cavity optomechanics at optical frequencies^14, 15^. As a result, the cantilever is only sensitive to the value of the electric energy <*W*_eTHz_> averaged over a terahertz oscillation cycle. The latter can be expressed as <*W*_eTHz_> = *P*_THz_ *Q*_THz_/2*ω*_THz_, where *P*_THz_ is the terahertz power dropped in the SRR. Then, according to Eq. (1), the net mechanical effect of a continuous terahertz wave is to displace the rest position of the cantilever. This effect can be resonantly enhanced if the incident terahertz intensity is modulated close to the cantilever mechanical frequency. For a sinusoidal modulation *P*_THz_ = $P_{{\mathrm{THz}}}^0$(1 + cos(*ωt*))/2, we can define a frequency-dependent internal responsivity of the system as the ratio between the amplitude of the resulting forced mechanical motion *y*(*ω*) and the peak terahertz power $P_{{\mathrm{THz}}}^0$ coupled into the SRR:

2 $$R_{{\mathrm{in}}}(\omega ) = \frac{{\left| {y(\omega )} \right|}}{{P_{{\mathrm{THz}}}^0}} = \frac{{Q_\alpha }}{{2m_{{\mathrm{eff}}}\omega _\alpha ^2}}\frac{{Q_{{\mathrm{THz}}}}}{{d_{{\mathrm{gap}}}^{{\mathrm{eff}}}\omega _{{\mathrm{THz}}}}}\left| {H_\alpha (\omega )} \right|$$

Here *H*_*α*_(*ω*) is the complex transfer function of the harmonic oscillator, normalized such as |*H*_*α*_(*ω*_*α*_)| = 1. We introduced the effective capacitive gap $d_{{\mathrm{gap}}}^{{\mathrm{eff}}}$, which depends on the details of the charge distribution at resonance. For our structure, we estimate $d_{{\mathrm{gap}}}^{{\mathrm{eff}}}$ ~ 800 nm based on simulations of the electrostatic energy as a function of the cantilever endpoint displacement *y* (Supplementary Note 1). Using Eq. (2) together with the value of the expression of the peak noise density of the Brownian motion, we evaluate the internal noise equivalent power (NEP) for this mechanism, defined as *P*_NEP_ = S_*yy*_(*f*_*α*_)^0.5^/*R*_in_(*f*_*α*_):

3 $$P_{{\mathrm{NEP}}} = \sqrt {2k_{\mathrm{B}}Tm_{{\mathrm{eff}}}\Gamma _\alpha } \frac{{2\omega _{{\mathrm{THz}}}d_{{\mathrm{gap}}}^{{\mathrm{eff}}}}}{{Q_{{\mathrm{THz}}}}}$$

Here Γ_*α*_ = *ω*_*α*_/*Q*_*α*_ is the linewidth of the mechanical mode. For the current geometry, we obtain a peak responsivity *R*_in_(*f*_*α*_) ~ 34 fm nW^−1^ and *P*_*NEP*_ ~ 16 nW Hz^−0.5^.

To compare the present device to other optomechanical systems^15^, we have also evaluated the frequency pull parameter *g*_om_ = *ω*_THz_/$d_{{\mathrm{gap}}}^{{\mathrm{eff}}}$ = 21 GHz nm^−1^ and the amplitude of zero-point mechanical motion *y*_ZPF_ = (*ћ*/2*m*_eff_ *ω*_*α*_)^0.5^ = 33 fm, which yield a single-photon optomechanical coupling *g*_0_ = *g*_om_ *y*_ZPF_ = 0.7 MHz. This value is commensurable with the mechanical frequency of the cantilever, which, in the quantum regime, would imply the possibility to resolve individual THz photons by recording the induced quantized mechanical displacement. Such non-demolition ponderomotive probe of electromagnetic energy was already considered in the early work of Braginsky and Khalili^24^, and while it has remained out of reach for optical and microwave optomechanical settings, it could seemingly be accessible in the THz domain.

### Experimental results and photothermal effect

**Fig. 3:**
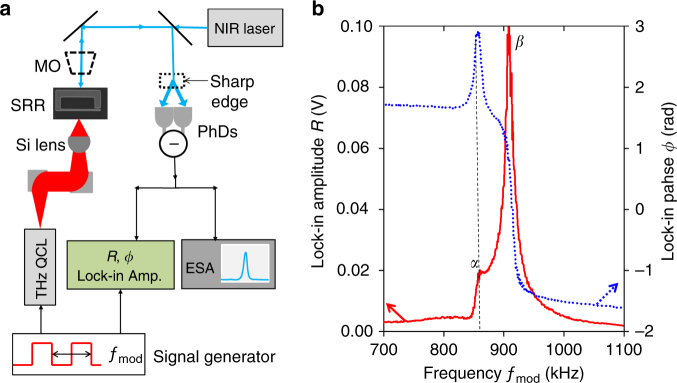
Terahertz optomechanical characterization. **a** Experimental setup of the terahertz optomechanical detection scheme. The near-infrared (NIR) laser is focused on the SRR through a microscope objective (MO). The laser beam reflected by the cantilever is divided into two parts by a sharp edge plate and sent to a balanced photodetector unit (PhDs). At the same time, the SRR is excited by a THz quantum cascade laser (QCL) with a current modulated by a signal generator at a frequency *f*_mod_. The signal from the PhDs is either monitored by an electronic spectrum analyser (ESA) or sent to a lock-in amplifier using a square wave reference with a frequency *f*_mod_. **b** Amplitude (full curve) and phase (dotted curve) from the lock-in, obtained as the modulation frequency of the QCL is swept around the mechanical resonances *α* and *β*. In this experiment, the Brownian motion spectrum monitored by the ESA is identical to the one of Fig. 2a. The dashed line indicates the position of the *α* resonance for clarity

The full experimental optomechanical setup, combining both mechanical read-out and THz excitation is reported in Fig. 3a. As a THz source, we use a quantum cascade laser (QCL)^25^, with an emission frequency of 2.6 THz and a maximum calibrated emitted power of 4.7 mW. The THz radiation from the QCL is collected with two parabolic mirrors and is focused on a single SRR with the help of a silicon hyperhemispherical lens^26^, positioned on the backside of the GaAs substrate. The THz QCL is either driven in pulsed mode, or in continuous wave with its current modulated with a signal generator at the frequency *f*_mod_.

The coherent mechanical response of the SRR induced by the THz radiation is studied by sweeping the QCL modulation frequency *f*_mod_ and recording both amplitude and phase on the lock-in amplifier connected to the PhDs. Figure 3b shows the response around the fundamental mechanical modes *α* and *β*. As can be seen, an important fraction of signal is collected not only from the in-plane mode *α* driven by the Coulomb force, but also from the out-of-plane mode *β*, which in the present experimental configuration has a larger response. The latter cannot be excited efficiently by the Coulomb force since the out-of-plane variation of the capacitance is negligible in the present geometry. Instead, the out-of-plane *β* mode is efficiently excited by a photothermal force arising from the bi-layer structure of the cantilever^22^. Indeed, in our device the incoming THz radiation generates heat through the eddy currents resonantly excited in the SRR, as illustrated in Fig. 1d. Since the GaAs and Au layers have different thermal expansion coefficients^22^, the temperature stress, also modulated at *f*_mod_, leads to the resonant excitation of the *β* mode. The photothermal force induced by the THz eddy currents can be modelled by solving the dynamical heat-transfer equation in the harmonic regime and by calculating the thermally induced stress, as outlined in Supplementary Note 2. As the resulting *z*-displacement of the cantilever is proportional to the absorbed THz power, we ultimately obtain the photothermal responsivity:

4 $$R_{{\mathrm{int}}}^{{\mathrm{ph}}}(\omega ) = \frac{{\left| {z(\omega )} \right|}}{{P_{{\mathrm{THz}}}^{\mathrm{0}}}} = \frac{{f_{{\mathrm{ph}}}^{\mathrm{0}}Q_\beta }}{{m_{{\mathrm{eff}}}\omega _\beta ^2}}\left| {Y(\omega )H_\beta (\omega )} \right|$$

5 $$Y\left( \omega \right) = \mathop {\sum}\limits_{n = 0}^\infty {\frac{{A_n}}{{i\omega \tau _0 + \left( {2n + 1} \right)^2}}}$$

Here $f_{{\mathrm{ph}}}^{\mathrm{0}}$ is the amplitude of the photothermal force per absorbed unit THz power, which is a function of the bi-layer geometry and material physical constants (Supplementary Note 2), *n* is an integer, *A*_*n*_ is a series of dimensionless-coefficients that depend on the spatial profiles of the eddy currents, the temperature rise and the shape of the cantilever mechanical mode. The quantity *H*_*β*_(*ω*) is the transfer function of the harmonic oscillator *β*. Values and more details are provided in Supplementary Note 2. The time constant *τ*_0_ is expressed as *τ*_*0*_ = 4*τ*/*π* ^2^, where *τ* = *L* ^2^/*D* is the thermal diffusion time (*D is* the diffusion coefficient provided in Supplementary Note 2), which in the present geometry is *τ* = 3 µs. The function *Y*(*ω*) defined in Eq. (5) takes into account the retardation effects of heat propagation along the cantilever, and basically acts as a low pass frequency filter on the instantaneous harmonic oscillator response *H*_*β*_(*ω*)^27^. For the *β* mode, Eq. (4) provides a peak responsivity of 860 fm nW^−1^, i.e. ~25 times larger than the response for the Coulomb mechanism associated to the *α* mode *R*_in_(*f*_*α*_), which explains quantitatively our experimental results.

### Comparison between photothermal and Coulomb forces

**Fig. 4:**
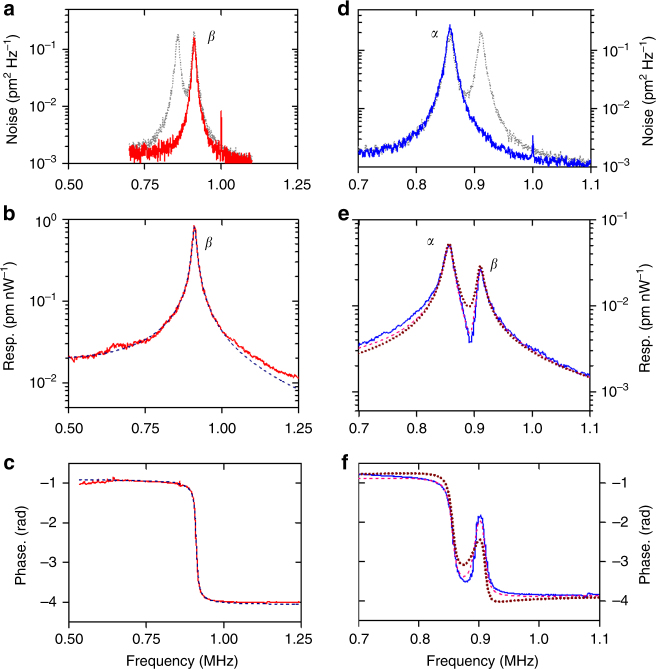
Experimental data and modelling. **a** Radio frequency (RF) spectrum with near-infrared (NIR) detection optimized on the *β* resonance (red curve). The dotted curve reproduces the spectrum from Fig. 2a. **b**, **c** Lock-in amplifier amplitude (**b**) and phase (**c**) measured in this condition (continuous curve). The dashed curves are computed from Eq. (4). **d** RF spectrum with NIR detection optimized on the *α* resonance (blue curve). **e**, **f** Corresponding amplitude (**e**) and phase (**f**) (continuous curves). Dotted curves: fit assuming that the force acting on the *α* resonance is described by Eq. (2), while the force acting on the *β* resonance is described by Eq. (4). Dashed curves: fit including a photothermal contribution to the optomechanical force acting on *α*

A key feature of our NIR detection scheme is that the overall signal is a linear superposition of in-plane and out-of-plane modes, |*I*_*y*_ *y*(*t*) + *I*_*z*_ *z*(*t*)|, where the coefficients *I*_*y*,*z*_ are real and can be adjusted by changing the position of the focal point of the NIR laser on the cantilever, as illustrated in Fig. 4. This allows favouring the detection of in-plane or out-of-plane modes, in order to study the corresponding THz response mechanisms. The coefficients *I*_*y*_ and *I*_*z*_ are directly provided by fitting the RF Brownian spectra, as shown in Fig. 2a (Methods), which corresponds to a particular case *I*_*y*_ = *I*_*z*_ = 0.5.

In the data presented in Fig. 4a–c, the detection of the out-of-plane *β* mode has been favoured in the Brownian spectra, *I*_z_ = 1 ≫ *I*_y_. The THz optomechanical response, measured with the lock-in amplifier, is shown in Fig. 4b (amplitude) and Fig. 4c (phase). The data are very well fitted by the model from Eq. (4) (dashed curves), assuming that only the *β* mode is present, setting the absolute values of the peak responsivity and phase. The peak responsivity of the *β* mode was quantified experimentally by using the calibration of the QCL output power (4.7 mW) and the maximum mechanical displacement of the cantilever (*z*_max_ = 71 nm), in the case were the QCL was modulated with a 16.5% duty-cycle square wave. This yielded an external responsivity of 50 fm nW^−1^, which is compatible with the value 860 fm nW^−1^ estimated by our model when considering 5% coupling efficiency of the silicon lens (Methods). The corresponding internal NEP is estimated at 0.4 nW Hz^−0.5^, which is already close to the state of the art of Golay cells^12, 13^. Note that, while leading to a photothermal force, the QCL heating in our structure does not alter the detection noise floor. Indeed, our model allows estimating a temperature rise at best on the order of 0.1 K, for a maximum QCL laser power of 4.7 mW impinging on the device (and 14.5% of square wave duty cycle modulation). Therefore the laser heating does not significantly change the Brownian noise at 300 K, which limits the noise equivalent power of our device in the present configuration.

In contrast, for the data presented in Fig. 4d–f, the detection of the *α* in-plane mode is favoured over *β*. Even though the *β* resonance is no longer visible in the RF spectra (Fig. 4d), it still contributes significantly to the forced oscillations induced by the THz radiation, as shown in Fig. 4e. Furthermore, the phase shown in Fig. 4f displays a peculiar feature around the *β* resonance. A first attempt to fit the data is done by assuming that only Coulomb instantaneous force acts on the in-plane mode *α* (Eq. (2)), while only a retarded photothermal force acts on the *β* mode Eq. (4)). This is shown by the dotted lines in Fig. 4e, f. Even though a good agreement is found, the amplitude of the phase variations in-between the two resonances is not reproduced exactly. A better fit (dashed curves in Fig. 4e, f) is obtained by adding to the instantaneous Coulomb force exciting the *α* mode an additional retarded photothermal contribution of the form *C*_ph_ *H*_*α*_(*ω*)/(1 + i*ωτ*), as described in the model of ref. ^27^. Here the constant *C*_ph_ is of similar magnitude as that obtained for the Coulomb force. Such effect can arise from the residual strain of the cantilever and the asymmetry of the clamping (Fig. 1a). Note that the delay factor *Y*(*ω*) is essentially an imaginary number around the *β*-resonance, and therefore we expect a phase shift close to *π* in that case. Instead, the instantaneous force acting on the *α*-resonance is characterized by a *π/*2 shift at resonance. Therefore, our analysis was aided by the presence the photothermally induced *β* -resonance in Fig. 4e, f, providing a phase reference. Finally, in the hypothesis that the THz force exciting the *α* mode is of purely photothermal origin, the phase behaviour is drastically different from Fig. 4e (Supplementary Fig. 6), unambiguously confirming the presence of an instantaneous Coulomb contribution to the optomechanical force.

## Discussion

In our device, both the instantaneous Coulomb force and the photothermal force arising from the bilayer structure can be used for fast terahertz detection at room temperature. Even though in the present geometry the Coulomb force has smaller amplitude than the force from the bilayer, it has the net advantage of not being limited by the thermal diffusion time constant *τ*. Indeed, *P*_NEP_ provided by Eq. (3) is independent from the mechanical frequency. Our resonator concept, which allows localizing the eddy currents and heat generation into very small volumes, also allows reducing the thermal diffusion time, i.e. by reducing the cantilever dimensions^28^, potentially leading to times on the order of nanoseconds. Unlike other fast room temperature terahertz detectors^11^, our structure presents engineering degrees of freedom that are free from material parameters. Improved photon collection can be achieved through integrating our resonators with planar antennas^10^, and *P*_NEP_ below the 100 pW Hz^−0.5^ level becomes achievable. Both detection mechanisms are already suitable for room temperature applications with externally modulated sources and they allow detecting at frequencies that are much higher than those of conventional terahertz detectors. The planar geometry of our structure is also very convenient for large-scale integration in imaging arrays^5^, or for integrating the device on a single chip.

In conclusion, we have introduced a metamaterial resonator where the strong sub-wavelength confinement allows converting electromagnetic energy into micromechanical motion at the nanoscale. Besides the dissipative photothermal forces, we have demonstrated a THz reaction (Coulomb) force that realizes the fundamental Hamiltonian of quantum opto-mechanics^15^. The Coulomb force can be optimized by increasing the quality factors of both THz and mechanical resonators. For instance, higher electromagnetic quality factors *Q*_THz_ up to 100–200 are achievable in symmetric resonators^29^. When tested in vacuum (data not shown), the present cantilevers displayed mechanical quality factors on the order of *Q*_*α*,*β*_ = 1000–3000. Furthermore our structure is based on semiconductor technology and operates in a frequency range where THz electronic transitions in quantum heterostructures can be achieved^8, 30^. Therefore, beyond the detector application, our device concept could open new perspectives for fundamental studies of optomechanical and light–matter interactions in the THz range.

## Methods

### Sample fabrication protocol

The composition of the wafers used to fabricate our structures is the following: a semi-insulating GaAs substrate, an epitaxially grown GaAs 320 nm buffer layer, a 1.6 µm thick Al_0.8_Ga_0.2_As sacrificial layer and a 320 nm thick top GaAs layer. The structures are defined by electron beam lithography on PMMA 950k resist. After exposing the resist, a layer of 5 nm of platinum (Pt) and 150 nm of gold (Au) are evaporated on the sample. Then, a lift-off is performed in order to define the metallic patterns that serve as an etch mask for dry etching in an inductively coupled plasma (ICP) reactor. In this step, the top GaAs and Al_0.8_Ga_0.2_As sacrificial layers are removed everywhere, but under the features protected by the metal. The sample is then wet-etched in a refrigerated (4 °C) dilute 2.5% hydrofluoric acid solution for 45 s, which allows removing anisotropically the sacrificial layer of Al_0.8_Ga_0.2_As with a depth to lateral etching ratio of 1.3. To avoid the sticking of the cantilever to the substrate, the sample is put on a hot plate at 200 °C for 60 s.

### THz spectroscopy of split-ring resonators

To infer the THz resonances of our split ring resonators, we have recorded transmission spectra with a Fourier transform interferometer (FTIR). The schematics of the experimental setup are shown in Supplementary Fig. 1a. Radiation from the globar source of the FTIR was focused and collected after passing through the structure using a planar mirror and three *f*1 parabolic mirrors, and finally detected by a cooled Germanium bolometer. A polarizer positioned at the output of the FTIR allowed selecting the polarization of the incident beam. The whole setup is placed in a purged environment in order to minimize water absorption. Measurements were performed on a dense array of resonators, as shown in Supplementary Fig. 1b, with 5 µm spacing between the structures, in order to obtain clear signatures of the resonances in the transmission spectra^9, 23^. Indeed, the typical beam spot size was of the order of 1 mm^2^, i.e. much larger than the typical cross section of a single resonator (4 × 10^−4^ mm^2^). Spectra were normalized using a reference spectrum obtained from the transmission through the bare semiconductor substrate. A baseline correction was also applied.

In Fig. 1b of the main text, the SRR resonance appears as a transmission dip at 2.7 THz. The position of the resonance agrees well with the formula: *ω*_THz_ = *πc*/*n*_eff_ *P*, where *c* is the speed of light, *P* = 44.5 µm the perimeter of the resonator in Fig. 1a, and *n*_eff_ is an effective index. This formula assumes a *λ*/2 standing wave pattern with electric field antinodes at the plate sides of the SRR. Knowing the frequency *ω*_THz_/2*π* = 2.7 THz we obtain an effective index *n*_eff_ = 1.27. This value compares well to the value obtained with the formula *n*_eff_ = (1 + *r* + (1 − *r*)*n*_AlGaAs_)/2 = 1.21, where *r* is the ratio between the length of the cantilever (suspended part) and the perimeter *P*. The latter formula averages between the refractive index of air and the refractive index of the Al_0.8_Ga_0.2_As layer below the resonator, with a bulk index *n*_AlGaAs_ = 3.18.

### Detection of the cantilever motion

The cantilever motion is measured using an optical detection scheme based on a near infrared λ = 940 nm laser diode. As described in Fig. 3a of the main text, the laser beam is focused on the cantilever through a microscope objective (MO). The fraction of the NIR light reflected by the cantilever is split in two spatially separated beams using a sharp edge blade. These beams are subsequently focused on a balanced photo-detection unit connected to a spectrum analyser (SA). Generally speaking, the light intensity *I*(*y*, *z*) scattered by the cantilever is a function of its in-plane (*y*) and out-of-plane (*z*) positions. Our optical detection setup provides the intensity fluctuations induced by the cantilever displacements with respect to the rest point of the latter:

6 $$\delta I = \frac{{\partial I}}{{\partial y}}y + \frac{{\partial I}}{{\partial z}}z = I_{\mathrm{y}}y + I_{\mathrm{z}}z$$

Here *I*_*y*_ and *I*_*z*_ are real coefficients that depend on the position of the laser spot focused on the cantilever, and can be adjusted experimentally (Fig. 4a–d). As a result, the signal provided by the SA will be proportional to the linear combination *I*_*y*_ *S*_*yy*_(*f*) + *I*_*z*_ *S*_*zz*_(*f)*, where *S*_*yy*_ and *S*_*zz*_ are the spectral components of the Brownian noise power density of the cantilever displacements^15^. Furthermore, the total noise can be expressed as *γS*_*yy*_(*f*) + (1 − *γ²*)^1/2^ *S*_*zz*_(*f*) + *S*_0_(*f*), where *S*_0_ is the noise floor of the photodetectors, and *γ* = *I*_y_/($I_y^2$ + $I_z^2$)^1/2^. Since we can experimentally set *γ* = 0 or *γ* = 1, and the noise floor is constant with frequency and much lower than the Brownian noise, we can use the analytical expressions for *S*_*yy*_(*f*) and *S*_*zz*_(*f*) in order to calibrate the SA readings. Finally, we note that the situation where *γ* = 1/2 corresponds to the Boltzmann equipartition theorem.

In the full set-up including the externally modulated THz QCL (Fig. 3a), the output of the balanced detection was either sent to a spectrum analyser, or to a lock-in amplifier referenced to the QCL modulation frequency *f*_mod_.

### Mechanical characteristics of the cantilever

The cross section and material composition of our cantilever is indicated in Supplementary Fig. 2. As shown in Fig. 1a the length *L* = 15.7 µm of the cantilever is measured from the free end to the mid-point of the etched pedestal. Regarding the cantilever as a homogeneous object, the resonance of the first in-plane flexural mode is described by the equation *f*_*α*_ = 0.162(*w*/*L* ^2^)(*Y*/*ρ*)^[1/231^. Here *Y* is the Young’s modulus and *ρ* the volume density of the cantilever. Neglecting the 5 nm Pt adhesive layer indicated in Supplementary Fig. 2, the main constituents of the cantilever are gold (Au) and GaAs, with the following characteristics:

$$Au\left\{ {\begin{array}{*{20}{c}} {Y_{Au} = 79\,GPa} \\ {\rho _{Au} = 19.3g{\mathrm{/}}cm^3} \end{array}} \right.,GaAs\left\{ {\begin{array}{*{20}{c}} {Y_{GaAs} = 85.5\,GPa} \\ {\rho _{GaAs} = 5.3g{\mathrm{/}}cm^3} \end{array}} \right.$$

As the gold and GaAs have very similar Young’s moduli, we take the average *Y* = 0.5(*Y*_Au_ + *Y*_GaAs_) = 82.25 GPa. Similarly, we use a mean density averaged over the thickness of the different materials: *ρ* = (*ρ*_Au_ *t*_1_ + *ρ*_GaAs_ *t*_2_)/(*t*_1_ + *t*_2_) = 9.8 g cm^−^^3^. Using these values, we obtain a numerical estimate *f*_*α*_ = 845 kHz, which compares very well with the experimental value 860 kHz, considering the fact that dimension are known with 10% uncertainty. Similarly, the frequency of the out-of-plane mode is provided by the formula *f*_*β*_ = *f*_*α*_(*t*_1_ + *t*_2_)/*w* = 910 kHz, and it is in excellent accordance with the experimental value. This justifies our approach of using average density and Young's modulus for the cantilever.

The cantilever also has higher order modes that are visible in the RF spectrum of its room temperature Brownian motion, as shown in Supplementary Fig. 3a, b. The frequencies of the higher-order modes are obtained from the expression *f*_(*α*/*β*)*N*_ = 2.81 *f*_(*α*/*β*)_(*N* − 0.5)^2^, with *N* an integer, also in excellent agreement with the experimental values *f*_*α*2_ = 5.4 MHz and *f*_*β*2_ = 5.7 MHz from the data presented in Supplementary Fig. 3a, meaning that the cantilever geometry is known with high precision.

The effective mass *m*_eff_ of the cantilever is obtained according to the formula *m*_eff_ = (33/140)*ρw*(*t*_1_ + *t*_2_) = 8.5 pg^31^. This mass corresponds to the endpoint movement of the cantilever, and we checked numerically that it is constant for all flexural modes. The frequency can thus be re-expressed as *f*_*α*_ = (1/2*π*)(*k*/*m*_eff_)^1/2^, where *k* = 0.25 N m^−1^ is the effective spring constant (similar for both fundamental modes). Using the parameters *k* and *m*_eff_ defined above, we can treat the cantilever as a one-dimensional harmonic oscillator. Within this picture, we can also express the noise spectral density of the Brownian motion of the cantilever S_*yy*_(*f*) as a function of frequency^15^, for instance for the *α* mode we obtain:

7 $$S_{{\mathrm{yy}}}(f) = \frac{{2k_{\mathrm{B}}T{\mathrm{\Gamma }}_\alpha }}{{m_{{\mathrm{eff}}}(2\pi )^3}}\frac{1}{{\left( {f^2 - f_\alpha ^2} \right)^2 + \left( {f{\mathrm{\Gamma }}_\alpha } \right)^2}}$$

A similar expression holds for *S*_*zz*_(*f*) and all other modes. Fitting the experimental spectra of the room temperature Brownian motion with the above equation allows, on one side, inferring the broadening parameter Γ for each mode, and, on the other side, calibrating the signal from the spectrum analyser, as described above and in the main text.

### Terahertz quantum cascade laser characteristics and power

The QCL used in this experiment has an active region similar to the one described in ref. ^25^. It was processed in a 3 mm-long single metal ridge waveguide. The QCL threshold current is *I*_th_ = 1.0 A, and the output power is of the order of a few mW at a heat sink temperature of 20 K. Using a calibrated THz power meter (Ophir 3A-P-THz ROHS), we rerecorded a maximum emitted power of 4.7 mW. In Supplementary Fig. 4, we show the spectrum of the QCL recorded at an applied bias of 48.5 V superimposed to the transmission spectrum of the SRR array from Supplementary Fig. 1b. The QCL has a central emission frequency of 2.6 THz, slightly detuned from the SRR resonance at 2.7 THz, still within the SRR resonant bandwidth.

To modulate the driving THz force on the cantilever, the QCL’s current was modulated from threshold to the maximum output power with a square wave with a duty cycle of 16.5%. In Supplementary Fig. 5, we show the time trace of the driving current (red curve) measured with an electronic oscilloscope. When the modulation frequency was matched to the mechanical frequency of the cantilever, the latter showed harmonic oscillations, as shown by the photocurrent from the balanced detection unit (blue curve), also recorded by an oscilloscope. This means that the mechanical resonator responds to the first Fourier harmonic of the square wave intensity modulation. The latter has an amplitude that is sin(0.16*π*)/*π* of the square wave amplitude, and was used for the experimental estimation of the detector noise equivalent power described in the main text.

To verify the resonant interaction between the THz source and the SRR, we also performed the detection experiment described in the main text using another QCL, which was emitting at 2.0 THz and was therefore detuned from the SRR resonance. The 2.0 THz QCL delivered a maximum power of 1 mW and was operated in the same conditions as the 2.6 THz QCL: pulsed mode with a 16.5%, duty cycle, and a modulation frequency matched to the cantilever mechanical resonance. As expected, using this laser we could not excite coherently the cantilever. Indeed, the QCL frequency being too far away from the SRR resonance, charge and current oscillations leading to a mechanical force on the cantilever could not be excited.

### Modelling of the residual thermal train of the in-plane mode

The residual thermal strain for the in-plane mode is modelled as described in ref. ^27^, that is as a force retarded by the thermal diffusion time *τ*. The frequency dependent expression of this force is then *C*_ph_/(1 + i*ωτ*)^27^. This is very similar to the photothermal force associated to the bilayer; indeed the function *Y*(*ω*) describes the retardation effects owing to the finite heat diffusion time across the cantilever length. However, no analytical expression for *C*_ph_ is known, and the latter is therefore treated as a fit parameter.

The modelling of the data in the hypothesis that only photothermal forces are present is shown in Supplementary Fig. 6. We first adjust *C*_ph_ so that the amplitudes of both the *α* and *β* modes are recovered (Supplementary Fig. 6a). The resulting phase, shown in Supplementary Fig. 6b, is very different from the measured one.

### Collection efficiency of the silicon lens

The numerical aperture of our lens was 0.60, providing a FWHM equal to 23 µm for the Airy spot inside the GaAs substrate of refractive index 3.5, for a THz QCL laser wavelength *λ* = 115 µm (2.6 THz). The corresponding area is 1640 µm^2^. The collection area of the SRR is estimated from the reflectivity data in Fig. 1b. The array unit cell in that case has an area of 308 µm^2^, and the resonance contrast is 0.35. This provides a collection area of 108 µm^2^ per resonator. The coupling efficiency is then estimated at 108 × 0.7/1640 = 4.6%, where the factor 0.7 is the air to Si transmission coefficient. Note that a larger collection area is expected for a single resonator as compared to a dense array^9, 23^.

### Data availability

The authors declare that the data supporting the findings of this study are available within the paper and its Supplementary Information files.

## Electronic supplementary material

Supplementary Information

Peer Review File
