# Supplementary material for: Optomechanical terahertz detection with single meta-atom resonator
Source: Nat Commun. 2017 Nov 17;8:1578. doi: 10.1038/s41467-017-01840-6 (PMC5691196; doi:10.1038/s41467-017-01840-6)
Supplement: Supplementary file 1 — Supplementary Information [file 41467_2017_1840_MOESM1_ESM.pdf]

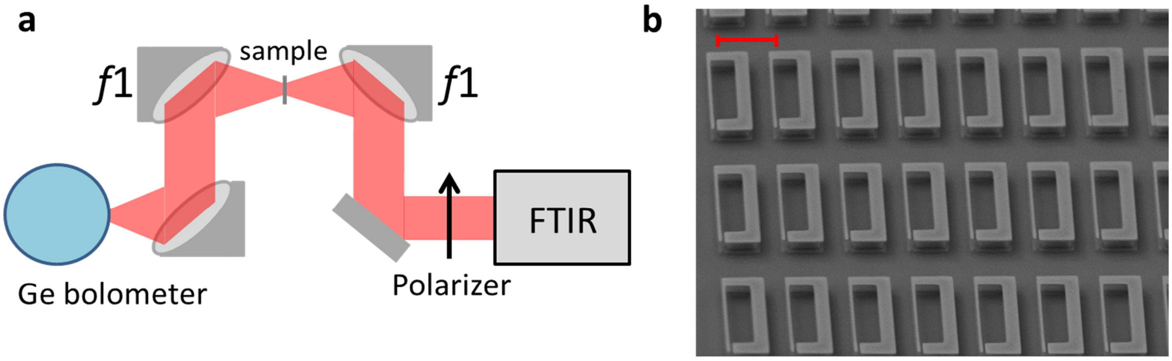

Supplementary Figure 1| Terahertz spectroscopy of split-ring resonators.
a Experimental setup used for the transmission measurements on the SRR samples. The beam from a far infrared Fourier transform interferometer (FTIR) is focused on the sample and then send to a germanium bolometer with the help of an optical mount that consist of a planar mirror and three *f*1 parabolic mirrors. A polarizer is placed at the exit of the FTIR. **b** Scanning electron microscope image of a dense array of identical resonators used for the transmission experiments. The scale bar placed in the upper left corner of the image is 10 μm wide.

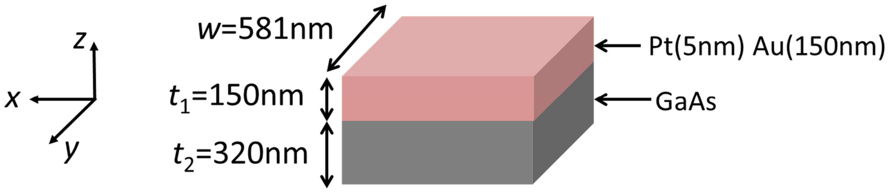

Supplementary Figure 2|Cantilever cross section.
Schematics of the cantilever cross section, indicating the materials of the bilayer and their thickness. There is a 5 nm thin adhesive platinum (Pt) layer between the 150nm gold (Au) and 320 nm GaAs layers; however this layer has negligible thickness and has not been taken into account in the models reported in the main text. A coordinate system has been placed next to the scheme in order to indicate the position of the layers with respect to Fig. 1a.

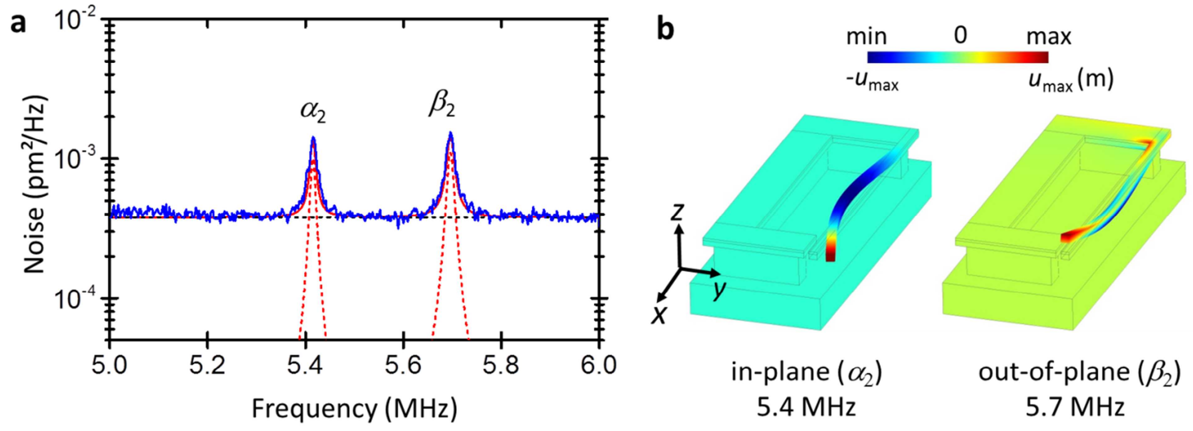

Supplementary Figure 3| Higher order mechanical modes.

a Blue curve**:** radio frequency spectrum of the second order in-plane (α_2) and out-of-plane (β_2) flexural cantilever modes. Red curve: fit resulting from the sum of the oscillator's spectral noise with the noise floor of the balanced photo-diode unit (black dashed curve). **b** Numerical modelling of the amplitude of deformation u_{max} for each mode.

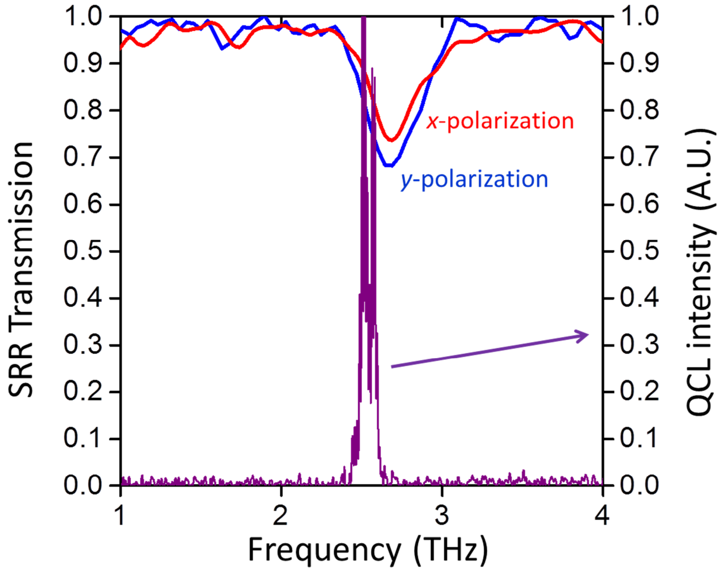

Supplementary Figure 4|Quantum cascade laser emission spectrum and SRR transmission.

Quantum cascade laser emission spectrum (magenta) superimposed to the transmission spectrum of the SRR arrays from Supplementary Fig. 1b. "*x*/*y*-polarization" refers to transmission measurements where the incident electric field is either along the SRR gap ("*y*"), or along the cantilever ("*x*"). See also Fig. 2b in the main text. In our experiments, the laser electric field was always along the *y*-direction.

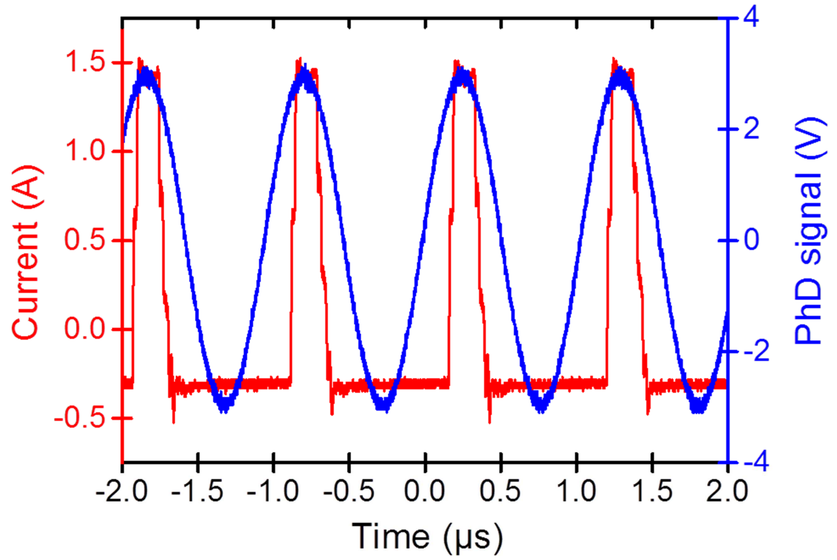

Supplementary Figure 5| Time traces of the cantilever oscillation.

Time traces representative of the incident THz power and the corresponding mechanical response of the cantilever. In red: quantum cascade laser driving current. In blue: signal from the balanced photo detection unit, recorded on an electronic oscilloscope.

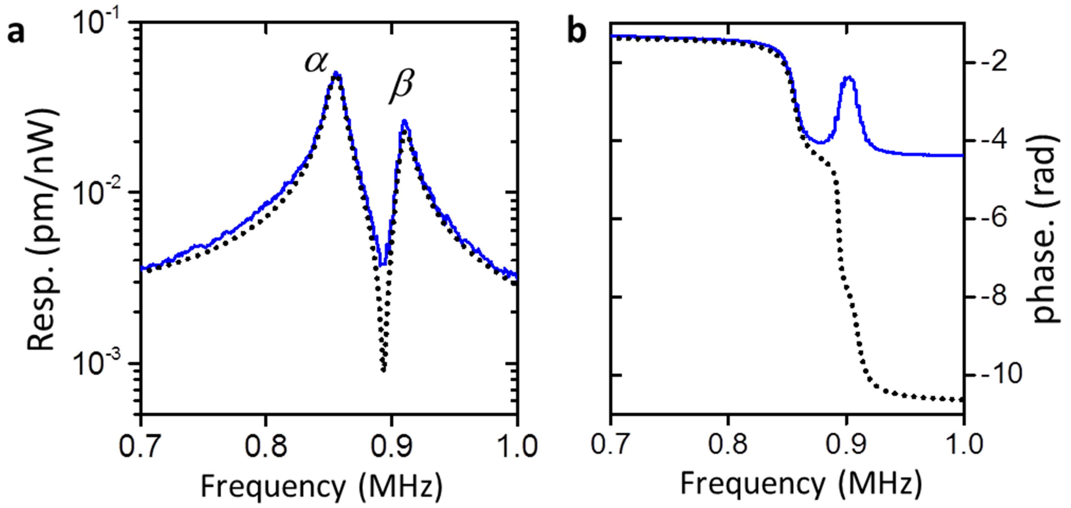

Supplementary Figure 6| Data modelling with purely photothermal forces.

Fit of the amplitude **a** and phase **b** of the same data as in Fig. 4e and Fig. 4f in the main text, but assuming purely photothermal forces for both α and β modes. The data is reproduced as continuous lines and the model is dotted curves.

Supplementary Note 1:
Terahertz optomechanical coupling and effective capacitance

The coupling between the SRR and the cantilever movement can be described in the picture of an equivalent inductance-capacitor circuit [1,2]. The electric field in the gap shown in Fig. 1a is described by an equivalent capacitor $C_{\text{eff}}(y)$ which is a function of the cantilever displacement y. Therefore, the dynamical variables of the coupled system are y, the displacement of the cantilever tip, and q, the charge induced on the capacitor plates. Then the system Lagrangian $\mathscr{L}$ is written as:

$$\mathscr{L} = \frac{1}{2} m_{\text{eff}} \dot{y}^2 + \frac{1}{2} L \dot{q}^2 - \frac{q^2}{2C_{\text{eff}}(y)} - \frac{1}{2} m_{\text{eff}} \omega_\alpha^2 y^2 \tag{1}$$

The cantilever movement can then be provided, for instance, by the Lagrange equation for the position:

$$m_{\text{eff}} \frac{d^2 y}{dt^2} + \frac{m_{\text{eff}} \omega_\alpha}{Q_\alpha} \frac{dy}{dt} + m_{\text{eff}} \omega_\alpha^2 y = \frac{d}{dy}\left(\frac{q^2}{2C_{\text{eff}}(y)} \right) \tag{2}$$

Usually, the displacement of the cantilever y is small compared to the gap d_{gap}, therefore the spatial derivative in the above equation can be approximated at zeroth order with its value at $y=d_{\text{gap}}$. Using the expression for the total electrical energy stored in the resonator $W_{\text{eTHz}} = q^2/2C_{\text{eff}}(y)$, and introducing an effective gap through the formula:

$$d_{\text{gap}}^{\text{eff}} = \left(\left. \frac{d \ln C_{\text{eff}}(y)}{dy} \right|_{y=d_{\text{gap}}} \right)^{-1} \tag{3}$$

we arrive at the results stated in the main text (Eq.(1)), with the function $H_\alpha(\omega) = (i\omega_\alpha^2 / Q_\alpha) / (\omega_\alpha^2 - \omega^2 + \mathrm{i}\omega_\alpha \omega / Q_\alpha)$. The parameter $d^{\text{eff}}_{\text{gap}}$ defined above allows evaluating the magnitude of the Coulomb force for an arbitrary geometry. However, its exact analytical evaluation is difficult, due to the fringing fields of the capacitance. To determine $d^{\text{eff}}_{\text{gap}}$ for our system we performed 2D quasi-static numerical simulations of the cross section of the structure in a plane perpendicular to the cantilever, using a finite element method. We evaluated the electric energy of the system W_{eTHz}, considering that the metallic part of the cantilever has a potential of 1 V, while the opposite metallic side is grounded. Performing such simulations for various positions y of the cantilever we obtained the effective gap $d^{\text{eff}}_{\text{gap}}$ = 800 nm. The electric field maps obtained in that case were very similar to the full electromagnetic simulation of the THz resonance mentioned in the main text. These simulations provided a negligible Coulomb force for cantilever displacements along the z-direction.

The fact that the effective gap (800 nm) is larger than the physical gap (308 nm, see Fig. 1a) is understandable, since the fringing fields which run away from the structure are expected to be less sensitive to the cantilever displacement compared to the field confined in the gap. Note

also that owing to propagation effects in the actual resonator the charge density develops along the whole cantilever length. Here we have considered only the charges in the vicinity of the gap, since the effects of the other parts are expected to be small as the distance between the positive and negative charges is much larger than d_{gap}.

Supplementary Note 2:
Modelling of the dynamic photothermal force induced by the eddy currents

The THz eddy currents shown in Fig. 1d induce an inhomogeneous heating described by a temperature profile $\Delta T(x)$, with the coordinate system O_{xyz} defined in Fig. 2b. Exceptionally, in this part z is a local coordinate and the cantilever displacement is denoted δz. The first step of our analysis is to determine the total elastic energy stored in the cantilever that corresponds to this heating effect. The elastic energy density can be expressed as $u(x,z) = -Y_i\gamma_i\Delta T(x)\varepsilon(x,z)$ [3] with i = 1 or 2 depending on whether z is in the Au or GaAs part, Y_i are the corresponding Young's moduli, γ_i the thermal expansion coefficients, and $\varepsilon(x,z)$ the deformation. Taking the deformation of a bent cantilever as $\varepsilon(x,z) = (z-z_0)/R(x)$, where $z_0 = (t_1+t_2)/2$ is the neutral axis and $R(x)$ is the local curvature radius, and by integrating over the cantilevers thickness we obtain for the total thermoelastic energy:

$$U = -wY\frac{(\gamma_1-\gamma_2)t_1t_2}{2}\int_0^L \frac{d^2\delta z}{dx^2}\Delta T(x)\mathrm{d}x \tag{4}$$

Here Y is the average Young modulus and we replaced the curvature with its approximate value $1/R(x) = \mathrm{d}^2\delta z/\mathrm{d}x^2$, the function $\delta z(x)$ describing the shape of the bent cantilever.

Actually, the thermal profile $\Delta T(x)$ depends not only on x but z as well. In the following, we determine $\Delta T(x,z)$ and we define an average over the cantilever thickness :

$$\Delta T(x) = \frac{1}{(t_1+t_2)}\int_0^{t_1+t_2} \Delta T(x,z)dz \tag{5}$$

that can be used directly with the expression for the thermoelastic energy in Supplementary Eq. (4).

To determine the full temperature profile we solve the Fourier heat equation in a steady state with a periodic heat source excitation at the top of the gold layer. This is justified as at THz frequencies heat is generated by the eddy currents within the skin depth of the metal. We first consider a harmonic temperature profile $\Delta T(x,z)\exp(i\omega t)$ that satisfies the heat equation:

$$\frac{i\omega}{D}\Delta T = \frac{\partial^2\Delta T}{\partial x^2} + \frac{\partial^2\Delta T}{\partial z^2} \tag{6}$$

With $D=(\lambda_1 t_1+\lambda_2 t_2)/(c_1\rho_1 t_1+c_2\rho_2 t_2)$ the thermal diffusion coefficient, λ_i the thermal conductivities and c_i the specific heat capacitances. The boundary conditions applied are: zero heat flow on the lateral surfaces of the cantilever and at its free end, except for the top gold surface. Since we consider only the dynamic part of the temperature profile we take $\Delta T = 0$ at the clamping point. The boundary condition on the gold surface is written: $-\lambda_1 \partial \Delta T / \partial z = P_{\mathrm{THz}}(x)/(L\Sigma)$ with $\Sigma=wL$ the top area of the cantilever. The function $P_{\mathrm{THz}}(x)$ is normalized such that $\int_0^L P_{\mathrm{THz}}(x)\mathrm{d}x$ is the total power P_{THz} dissipated by the eddy currents, which is basically the power coupled by the SRR. The solution of the heat equation with these boundary conditions is expressed by a series of cosine and hyperbolic functions. By averaging $\Delta T(x,z)$ over the cantilever thickness according to Supplementary Eq.(5) we obtain:

$$\Delta T(x) = \frac{-P_{tot}4L^2}{\lambda_1\pi^2\Sigma(t_1+t_2)}\sum_{n=0}^{\infty}\frac{p_n\sin\left(\frac{\pi x}{L}(n+0.5)\right)}{i\omega\tau_0+(2n+1)^2} \tag{7}$$

Here:

$$p_n = \int_0^L P(x)\sin\left(\frac{\pi x}{L}(n+0.5)\right)\mathrm{d}x / P_{THz} \tag{8}$$

are series of dimensionless coefficients describing the projection of the heat source on the spatial harmonics of the temperature profile. First, we include this expression in the formula of the elastic energy above. Second, we define a dynamical profile if the cantilever vibrational mode $\delta z(x) = z_{\max}Q_\beta(x)$, where $z_{\max}$ is the amplitude of the displacement of the free end of the cantilever, and $Q_\beta(x)$ is a dimensionless eigen-function which is a solution of the one-dimensional equation for elastic waves [3], normalized such that $Q_\beta(y) = 1$ at the free end. The total thermoelastic energy thus becomes:

$$U = \frac{2Y(\gamma_1-\gamma_2)t_1t_2}{\pi^2\lambda_1(t_1+t_2)}P_{THz}z_{\max}Y(\omega) \tag{9}$$

The function $Y(\omega)$ is defined in Eq.(3) of the main text with the coefficients $A_n=p_n r_n$. Here r_n are the dimensionless projection integrals:

$$r_n = L\int_0^L \frac{d^2Q_\beta}{dx^2}\sin\left(\frac{\pi x}{L}(n+0.5)\right)\mathrm{d}x \tag{10}$$

The expression of the effective photothermal force f_{ph} acting on the cantilever end is finally provided by:

$$f_{ph} = \frac{\partial U}{\partial z_{\max}} = \frac{2Y(\gamma_1 - \gamma_2)t_1 t_2}{\pi^2 \lambda_1 (t_1 + t_2)} P_{THz} Y(\omega) \tag{11}$$

Since f_{ph} is proportional to the absorbed power, we can define a force f^0_{ph} per unit absorbed THz power as in Eq.(4). For the models presented in this paper the absorbed power (Joule heating) is modelled with the spatial profile $sin^2(x\pi/L)$, which fits the current profiles provided by finite difference domain electromagnetic simulations illustrated in Fig. 1d.

Supplementary References

1. Andrews, R. W. *et al.* Bidirectional and efficient conversion between microwave and optical light, *Nature Phys.* **10**, 321–326 (2014).
2. Bagci, T. *et al.* Optical detection of radio waves through a nanomechanical transducer, *Nature* **507**, 81–85 (2014).
3. Landau L., & Lifchitz, E. Théorie de l'élasticité, (Editions Mir, 1967).
