# Supplementary material for: Optomechanical terahertz detection with single meta-atom resonator
Source: Nat Commun. 2017 Nov 17;8:1578. doi: 10.1038/s41467-017-01840-6 (PMC5691196; doi:10.1038/s41467-017-01840-6)
Supplement: Supplementary file 2 — Peer Review File [file 41467_2017_1840_MOESM2_ESM.pdf]

Editorial Note: this manuscript has been previously reviewed at another journal that is not operating a transparent peer review scheme. This document only contains reviewer comments and rebuttal letters for versions considered at Nature Communications.

REVIEWERS' COMMENTS:

Reviewer #2 (Remarks to the Author):

The authors replied to all my remarks in a convincing way.

I consequently feel they improved the technical quality of the manuscript significantly.

In my view, the manuscript can be now accepted for publication in Nature Communications

Reviewer #3 (Remarks to the Author):

After reviewing the revised manuscript, it appears the authors have improved the clarity of the manuscript and addressed my main concerns regarding placing this work in better context among other references on high bandwidth THz detection. I believe in its present form the manuscript is suitable for Nature communications. I also found the authors' response to the comments of reviewer #2 to be illuminating. In particular I would have liked to see more of the reply to "C3" regarding laser heating not significantly affecting the Brownian noise included in the text. I believe this might be a common source of confusion for readers when considering the discussion of photothermal forces.
